# Cooperative down-regulation of ribosomal protein L10 and NF-κB signaling pathway is responsible for the anti-proliferative effects by DMAPT in pancreatic cancer cells

**DOI:** 10.18632/oncotarget.16557

**Published:** 2017-03-25

**Authors:** Chen Shi, Yang Wang, Yuna Guo, Yijun Chen, Nan Liu

**Affiliations:** ^1^ State Key Laboratory of Natural Medicines and Laboratory of Chemical Biology, China Pharmaceutical University, Nanjing, Jiangsu Province, 210009, People's Republic of China

**Keywords:** DMAPT, RPL10, NF-κB pathway, mechanism, pancreatic cancer

## Abstract

Dimethylaminoparthenolide (DMAPT), a water-soluble analogue of natural product parthenolide, possesses anti-inflammatory and anti-tumor activities. Despite that the anti-inflammatory mechanism of DMAPT has been well studied, specific target(s) for DMPAT and its anti-tumor mechanism remain poorly understood. In this study, to assess the anti-proliferative effects of DMAPT in pancreatic cancer cell lines and exploit its anti-tumor mechanism, serial affinity chromatograph was implemented to probe potential targets for DMAPT, revealing that ribosomal protein L10 (RPL10) is a specific binding protein of DMAPT in PANC-1 cells. DMAPT could decrease the expression of RPL10 accompanying its anti-proliferative effects. Mechanistically, in both PANC-1 cells and MiaPaca-2 cells, reduced expression of RPL10 triggered by DMAPT binding decreased the expression of either p65 or IKKγ through the direct binding between RPL10 and p65 or IKKγ. Together, the present study strongly implies that RPL10 is a novel target with therapeutic potential for the treatment of pancreatic cancer.

## INTRODUCTION

Pancreatic cancer is a fatal disease with an overall 5-year survival rate less than 5% due to poor early diagnosis and lack of effective therapeutic options [1]. Each year, more than 330,000 patients are diagnosed with pancreatic cancer worldwide. Although tremendous efforts have been invested aiming to discover new therapeutic targets and effective agents for the treatment of pancreatic cancer, little progress has been achieved thus far [2–4].

**Figure 1:**
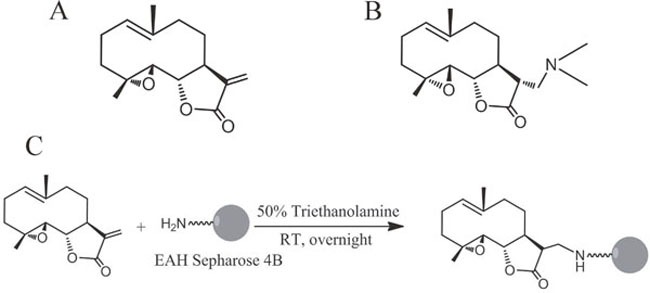
Chemical structures of PTL, DMAPT and DMAPT-affinity probe **(A)** Structure of PTL. **(B)** Structure of DMAPT. **(C)** Synthetic scheme of DMAPT-affinity probe.

Parthenolide (PTL, Figure 1A), a sesquiterpene lactone extracted from the shoots of feverfew (*Tanacetum parthenium*) [5], exhibits anti-inflammatory and anti-tumor activities [6]. PTL is a well-known NF-κB inhibitor owing to its ability of targeting several components of the NF-κB signaling pathway, such as the binding to NF-κB subunits [7] or the inhibition of IκB kinase (IKK) complex [8]. PTL also regulates MAPK and c-Jun-N-terminal kinase in melanoma cancer cells [9], and induces ROS generation, mitochondrial dysfunction and cell necrosis in triple-negative breast cancers cells [10]. Although PTL possesses potency against different types of cancers, the off-target effects, particularly high doses used and its strong hydrophobicity, are the major limitation for its potential clinical use [11]. Subsequently, a variety of water-soluble derivatives are prepared and evaluated. Among them, DMAPT (Figure 1B), bearing a dimethyl amino group, showed the most promise with improved pharmacokinetic properties including increased oral bioavailability [12], high plasma concentration following the administration and acceptable toxic profiles in animal models [13]. In mice, an oral dose of DMAPT at 100 mg/kg achieved a concentration maximum of 25 μM and a half-life of 0.63 h in serum [14].

Previous studies have shown that DMAPT can enhance the cytotoxic effects of gemcitabine in pancreatic cancer cells, such as BxPC-3 and MiaPaca-2 [15]. Meanwhile, DMAPT combined with gemcitabine remarkably decreases tumor size and the incidence of the metastasis to liver in mouse models of pancreatic cancer [16]. However, there is no generally accepted molecular target with regard to the anti-tumor activity of DMAPT up to date. Given the urgent need on diagnosis and treatment of pancreatic cancer and in an effort to discover molecular target(s) and elucidate the mechanism of action of DMAPT in pancreatic cancer, we confirmed the activities of DMAPT on four pancreatic cancer cell lines, and used chemical proteomics approach to probe its specific target in PANC-1 cells in the present study. Our results indicated that DMAPT specifically binds ribosomal protein L10 (RPL10), and the subsequent binding between RPL10 and either p65 or IKKγ resulted in reduced expression of these proteins in the NF-κB signaling pathway to exhibit the anti-proliferative effects in PANC-1 and MiaPaca-2 cells.

## RESULTS

### Confirmation of the sensitivity of DMAPT against pancreatic cancer cells

**Figure 2:**
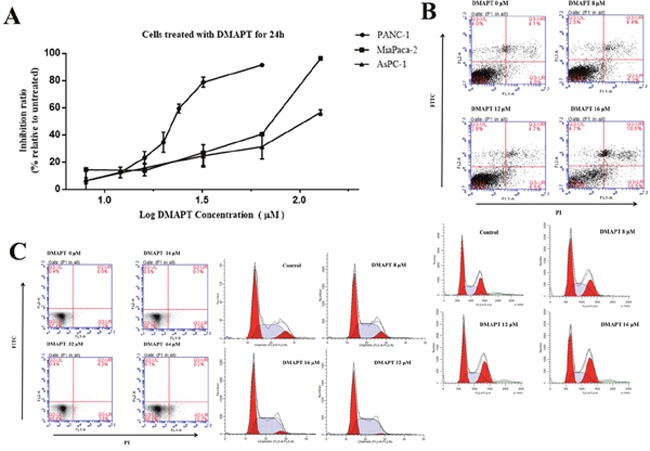
Effects of DMAPT on the viability of pancreatic tumor cells **(A)** Inhibition of the viability of pancreatic tumor cells by DMAPT. **(B)** Effects of DMAPT on apoptosis and cell cycle of PANC-1 cells. **(C)** Effects of DMAPT on apoptosis and cell cycle of MiaPaca-2 cells. Data are mean values of 3 independent experiments with standard deviations.

DMAPT inhibited pancreatic cancer cell viability in a dose-dependent manner with substantial inhibition observed in the range of 22 and 110 μmol/L in three cell lines (Figure 2). The PANC-1 cells were the most sensitive to DMAPT for its inhibition of cell viability (IC_50_ = 22.84 ± 1.38 μmol/L) compared to MiaPaca-2 cells (IC_50_ = 52.55 ± 0.63 μmol/L) and AsPC-1 cells (IC_50_ = 110.79 ± 0.6 μmol/L). On contrary, only 49.00% of cell viability could be inhibited when up to 128 μM DMAPT was treated to BxPC-3 cells. Meanwhile, the treatments of the cells by 12 μM DMAPT increased the spontaneous apoptotic level of PANC-1 cells from 4.1 ± 0.5% annexin V-positive cells to 10.5 ± 0.4% (p<0.01). (Figure 2B & Supplementary Figure 1). In addition, DMAPT significantly increased the number of PANC-1 cells in the S/G2 phase from 63.4 ± 7.6% to 83.57 ± 2.3% (p<0.001) by flow cytometry, indicating that DMAPT arrests the cell cycle of PANC-1 in S/G2 phase. On the other hand, little apoptosis was shown in MiaPaca-2 cells following the treatment with DMAPT of 64 μmol/L (Figure 2C & Supplementary Figure 1).

### Identification of cellular target of DMAPT

**Figure 3:**
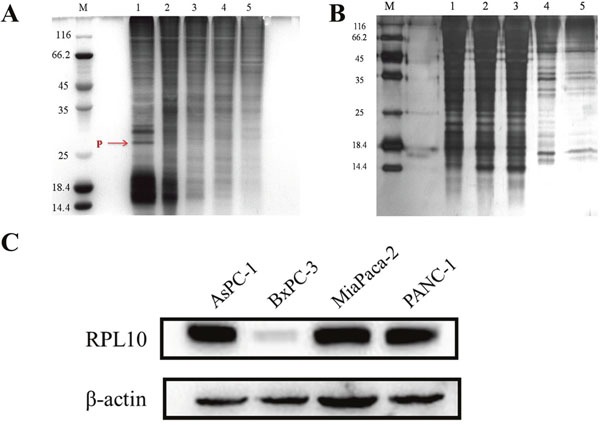
RPL10 specifically bound DMAPT **(A)** SDA-PAGE for serial affinity chromatography by silver staining. Lane M, protein molecular weight markers; Lane 1-5, resin-bound protein from 1-5 affinity series. Specifically bound protein is indicated in red arrow. **(B)** SDS-PAGE for blank matrices. Lane M, protein molecular weight markers; Lane 1-5, supernatants from 1-5 series of affinity chromatography. **(C)** The expressions of RPL10 in four pancreatic tumor cell lines.

To identify potential targets of DMAPT, an affinity probe was prepared by coupling the α-methylene-γ-lactone ring of PTL with EAH-sepharose 4B resins through a Michael addition. Owing to the similar structure between PTL-NH2-Sepharose 4B and DMAPT, the PTL-NH2-Sepharose 4B resins could represent the chemical nature of DMAPT and thus be used as an affinity probe. Compared to non-specific bindings with blank matrices (Figure 3A), a protein decreased in amount by the affinity probe was obviously found on SDS-PAGE after serial affinity chromatography (Figure 3B), suggesting that this band may represent a specific binding protein and potential target for DMAPT. The protein band was then excised from the gel for tryptic digestion and MALDI-TOF-MS analyses. Based on molecular weights of the tryptic peptides, MS results unambiguously revealed that the binding protein is RPL10, also named as QM (Supplementary Figure 2). Given that RPL10 exhibits extra-ribosomal functions, such as transcriptional and autogenous regulation of translation [17–18], the present finding on the specific binding between the affinity probe and RPL10 is not unusual. Meanwhile, the expression profiles of RPL10 in these pancreatic cancer cell lines were detected by Western blotting. PRL10 was found to obviously express in PANC-1, MiaPaca-2 and AsPC-1 cells, respectively (Figure 3C), whereas its expression level in BxPC-3 cells was almost undetectable. Thus, the expression of RPL10 seemed to show a positive correlation to the inhibitory activity of DMAPT against various pancreatic cancer cells.

### Verification of direct interaction between DMAPT and RPL10

**Figure 4:**
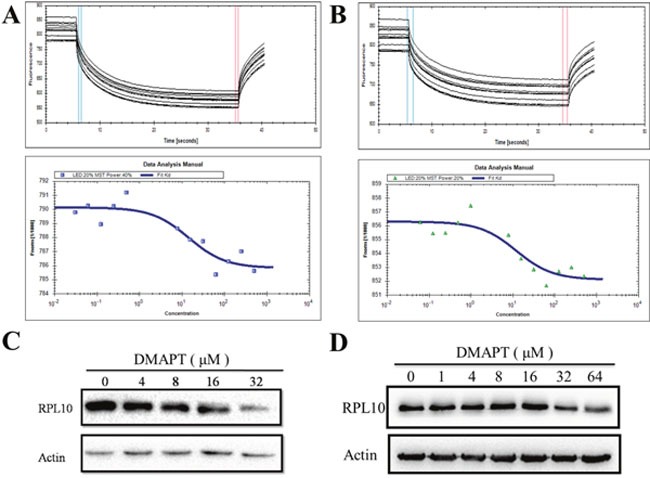
Interaction between DMAPT and RPL10 *in vitro* **(A)** Up panel: MST time traces of 12 concentrations of DMAPT. The core domain of RPL10 was mixed with the increase of DMAPT concentration. Thermodiffusion was amplified with increasing DMAPT concentrations; down panel: the dissociation constants of DMAPT to fluorescently labeled core domain of RPL10. **(B)** Up panel: MST time traces of 12 concentrations of DMAPT. Full-length RPL10 was mixed with increasing DMAPT concentration. Thermodiffusion was amplified with increasing DMAPT concentrations; down panel: the dissociation constants of DMAPT to fluorescently labeled full-length RPL10. **(C)** Inhibition of RPL10 expression by DMAPT in PANC-1 cells. **(D)** Inhibition of RPL10 expression by DMAPT in MiaPaca-2 cells.

To ascertain whether DMAPT directly interacts with RPL10, the binding affinity of DMAPT with RPL10 was measured using MST, a robust method to determine binding affinities between macromolecules and small molecules. Indeed, DMAPT could bind the core domain of RPL10 with a *K*_D_ value of 14.3 ± 2.04 μM (Figure 4A). In the case of full-length RPL10 and DMAPT, a *K*_D_ value of 11.9 ± 1.88 μM was obtained (Figure 4B). The MST results demonstrated that DMAPT is able to directly bind RPL10 protein, and the potential binding site could be within the core domain of RPL10.

Next, to examine how RPL10 mediates the effects of DMAPT in PANC-1 cells or MiaPaca-2 cells, treatments of PANC-1 cells or MiaPaca-2 cells with DMAPT for 24 h resulted in the decrease of RPL10 in a dose-dependent manner (Figure 4). In addition, IC_50_ value for DMAPT in PANC-1 cells after knock-down of RPL10 was increased to 49.63 μM, a 2-fold increase compared to the cells with normal RPL10 expression (Supplementary Figure 3). Together, these data further suggested that RPL10 is a specific and direct target of DMAPT.

### The interaction of RPL10 with NF-κB signaling pathway influenced by DMAPT

**Figure 5:**
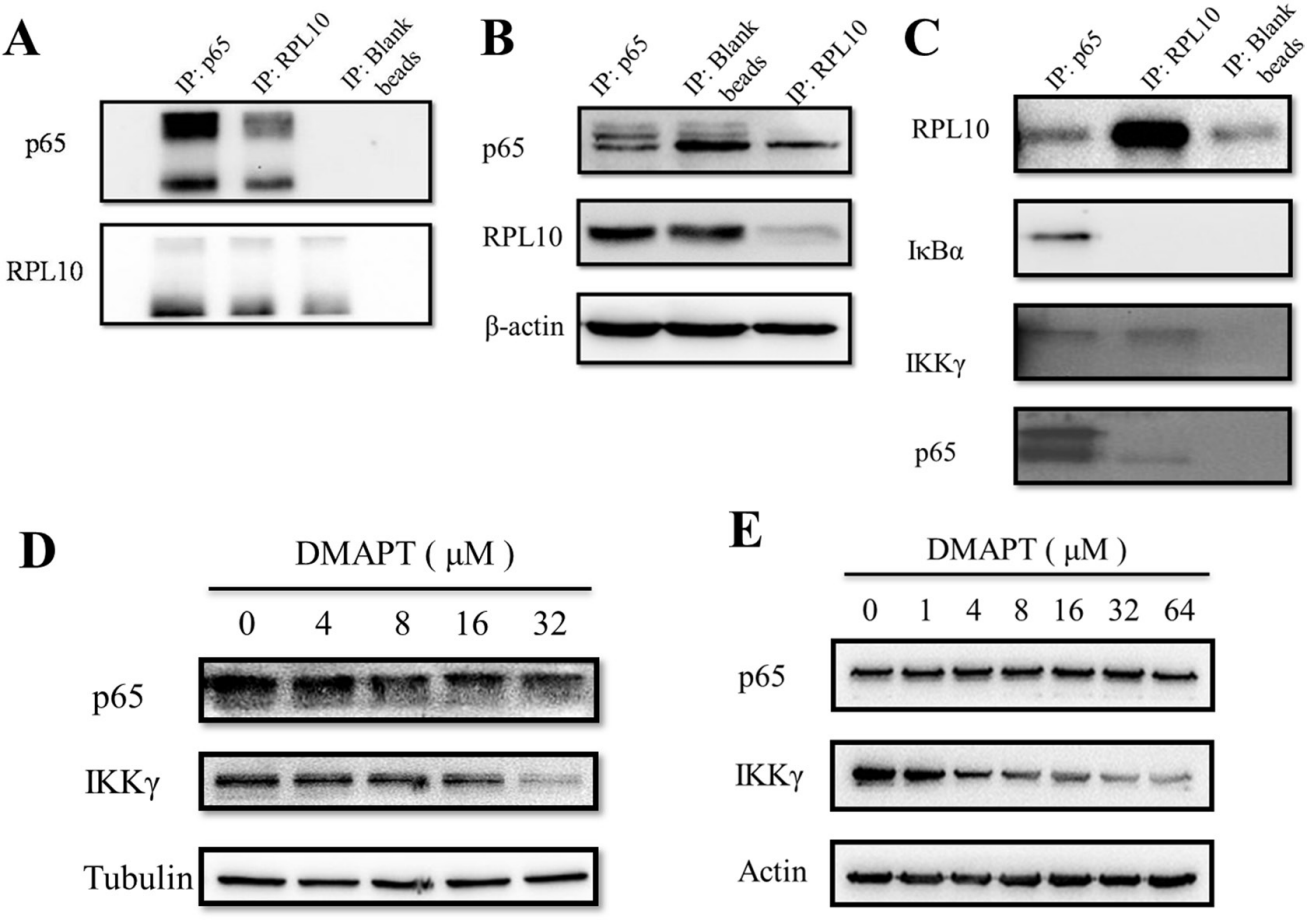
RPL10 interacted with p65 to decrease its expression **(A)** Interaction between RPL10 and p65 in PANC-1 cells. Left, agarose beads coupled with anti-p65 antibody; middle, agarose beads coupled with anti-RPL10 antibody; right, agarose beads without antibody. **(B)** Left: knock-down of *p65* or *RPL10* in PANC-1 cells respectively. Lane 1, knock-down of *p65* by specific siRNA; lane 2, PANC-1 cells without siRNA; lane 3, knock-down of *RPL10* by specific siRNA. Middle and right, grayscale of bands are shown in left. **(C)** RPL10 interacted with different components of NF-κB. Left lane is a positive control of immunoprecipitated proteins by anti-p65 antibody. **(D)** Inhibition of p65 and IKKγ expression by DMAPT in PANC-1 cells. **(E)** Inhibition of p65 and IKKγ expression by DMAPT in MiaPaca-2 cells.

Previously, the major anti-tumor mechanism of DMAPT has been centered in the inhibition of NF-κB signaling pathway, especially the inhibitory activity of IKK complex to interfere the phosphorylation of IκB. Thus, the interaction of p65 and RPL10 was explored by immunoprecipitation in PANC-1 cells (Figure 5A). To rule out potential false positives from the Sepharose beads, *p65* and *RPL10* were knocked-down respectively, and then the expression of individual protein was detected by Western blotting. This time, p65 expression was decreased after knocking-down *RPL10* in PANC-1 cells, whereas the expression of RPL10 did not change with the decrease of p65 (Figure 5C). The results confirmed that RPL10 can down-regulate the expression of p65. To determine whether other components in the NF-κB signaling pathway could interact with RPL10 in PANC-1 cells, other members in the NF-κB pathway were examined by immunoprecipitation, in which the proteins captured by anti-p65 antibody were regarded as positive controls. In addition to p65, IKKγ could directly bind RPL10, suggesting that RPL10 acts on multiple components in the NF-κB pathway.

**Figure 6:**
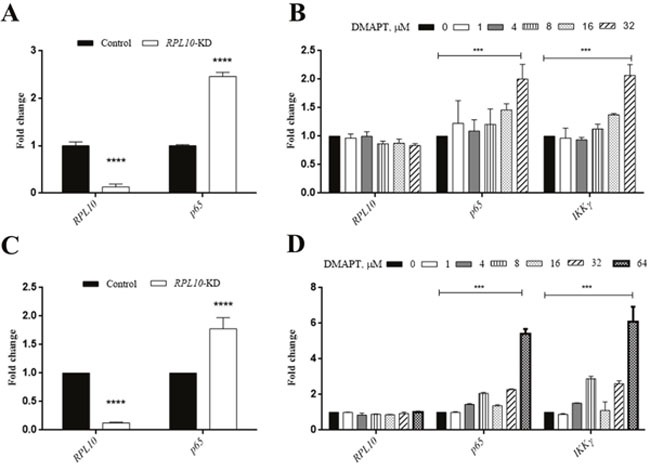
qRT-PCR confirmation of the interaction between *RPL10* and *p65* **(A)** Changes in mRNA level of *RPL10* and *p65* after knock-down of *RPL10* in PANC-1 cells. **(B)** Changes in mRNA level of *RPL10*, *p65*, *IKKγ* after DMAPT treatments of PANC-1 cells for 24 h. **(C)** Changes in mRNA level of *RPL10* and *p65* after knock-down of *RPL10* in MiaPaca-2 cells. **(D)** Changes in mRNA level of *RPL10*, *p65*, *IKKγ* after DMAPT treatments of MiaPaca-2 cells for 24 h.

To examine whether similar situation also occurred in MiaPaca-2 cells compared to PANC-1 cells, DMAPT could indeed lower the expression of p65 and IKKγ in a dose-dependent manner in both cell lines (Figure 6C). Next, to test the effects of DMAPT on gene transcription, mRNA levels of *RPL10* and *p65* in PANC-1 and MiaPaca-2 cells after the knock-down of *RPL10* were quantified. Notably, the increase of mRNA level of *p65* was found to opposite the change of its protein expression when *RPL10* was knocked-down, suggesting that the difference on p65 expression might be caused by the influence on translation step instead of transcription. Meanwhile, by the increase of DMAPT concentration, mRNA levels of *p65* and *IKKγ* were increased accordingly, which is in accordance to the knock-down of *RPL10*.

Previous studies indicated that STAT3 plays the principal roles in the anti-tumor function of DMAPT [14]. To examine the relationship between RPL10 and STAT3, immunoprecipitation showed that RPL10 was unable to bind STAT3 (Supplementary Figure 4), and the mRNA level of STAT3 was not affected by different concentrations of DMAPT as well, suggesting that the binding between DMAPT and RPL10 is not related to the STAT3 signaling pathway.

**Figure 7:**
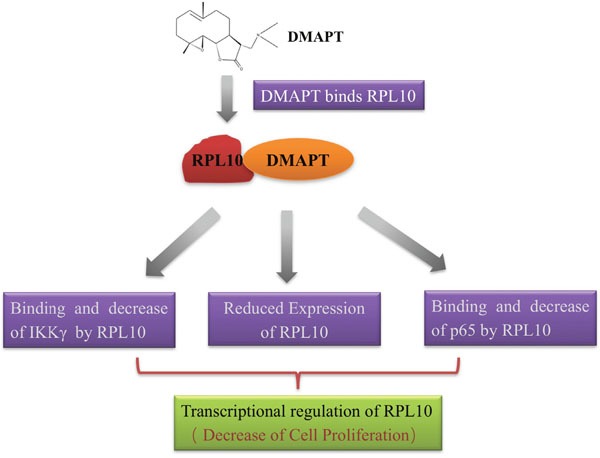
Proposed mechanism of action of DMAPT

Based on the experimental evidence in the present study, the anti-proliferative effects by DMAPT in pancreatic cancer cells are most likely the results of a synergetic action of RPL10 and its influence on the NF-κB pathway, which is illustrated in Figure 7. After the binding between DMAPT and RPL10, the expression of RPL10 is decreased, promoting the reduction of expression of p65 or IKKγ from the direct binding by RPL10, and then leading to the inhibition of the NF-κB pathway and consequent decrease of cell viability.

## DISCUSSION

The advancements in molecular and targeted therapies have greatly improved survival of cancer patients. However, the treatment outcome for pancreatic cancer has not changed much over the past three decades. The current standard treatments are the use of gemcitabine [19]. Unfortunately, it only results in very modest increases in median life expectancy due to the onset of chemo-resistance. The lack of effective therapy has motivated the search for new ways of combating this aggressive malignancy. In the present study, we showed that DMAPT exhibits significant anti-tumor activity in different pancreatic cell lines through inhibiting cell growth, inducing cell apoptosis and arresting cell cycle.

Previously, 10 μM DMAPT combined with gemcitabine has shown to produce anti-pancreatic cancer activity against PANC-1, MiaPaCa-2 and BxPC-3 cell lines [20]. However, the specific target and mechanism for DMAPT are largely unclear. The present study revealed that RPL10 can specifically bind DMAPT in PANC-1 cells by chemical proteomics and MST measurements. Although chemical proteomics is able to capture proteins that are capable of binding small molecules, the existence of non-specific binding proteins has been a major obstacle. Therefore, a comprehensive understanding of the protein-small molecule interaction requires the validation to associate their cellular functions and provide direct evidence on the specific interaction. In the present study, in addition to the investigation of functional consequences in cells, MST was used to measure binding affinity between the target protein and the small molecule. Compared to high affinity of antibody and antigen interaction, the binding affinities of DMAPT to the core-domain RPL10 and full-length RPL10 were relatively weaker, which is a common characteristic for small molecule-protein interaction due to lower sensitivity from the instrument limitations [21–22].

PTL is a well-studied NF-κB inhibitor, owing to its ability to target several components of the NF-κB pathway. The most consistently reported anti-inflammatory mechanism by PTL is the direct alkylation of p65 subunit of NF-κB at residue cys38 to prevent it from the binding to DNA [23]. On the other aspect, the activation of NF-κB has been implicated in pancreatic tumorigenesis. Combination of PTL with sulindac synergistically inhibited cell growth and increased the levels of IκBα expression, showing a cooperative targeting of the NF-κB pathway [24]. Despite the reports that the anti-inflammatory and anti-tumor activities of DMAPT are similar to that of PTL, the distinction between PTL and DMAPT against pancreatic cancer cells has been less understood. Thus, the present study investigated the relationship between RPL10 and the key regulators in the NF-κB pathway through the comparison of the effects of DMAPT to elucidate precise signal pathway in PANC-1 and MiaPaca-2 cells. We found that RPL10 can specifically bind p65 and IKKγ to regulate the expression of these molecules in the process of translation, indicating that RPL10 can actually regulate the NF-κB pathway to synergistically determine the fate of various cellular processes. We also observed that the transcription levels and protein expressions of p65 and IKKγ are opposite when the cells are treated by DMAPT. Given the ribosomal nature of RPL10, the reduced translation of p65 and IKKγ might attribute to the dysfunction of ribosomal biogenesis. So far, the importance of ribosome function and translational control in tumorigenesis has been underscored although several oncogenes, such as *Myc* [25], and tumor suppressor genes, such as *PTEN* [26] and *p53* [27], have been linked to direct regulation of ribosome production and protein translation. In prostate cancer patients, reduced expression of RPL10 has been suggested to associate with early development of the cancer; however, high expression of RPL10 at later stages of cancer development could drive forward to a more aggressive phenotype [28]. More importantly, mutations in *RPL10* gene have been recently detected in cancer patients who are unrelated to any heritable diseases [29]. Nevertheless, although a number of studies have dealt with the molecular basis and functions of RPL10, the actual roles in various types and stages of cancer remain elusive. According to the present study, p65 and IKKγ are also able to bind RPL10 to direct the regulation of the NF-κB pathway and the cellular processes in general. In terms of pancreatic cancer, the potential target of RPL10 for the treatment of this disease may relate to the extraribosomal functions as the autogenous regulation of translation. RPL10 may bind certain mRNA in specific ribosomal protein cistrons and inhibit translation to regulating cancer progression. Additionally, RPL10 is an apparent Jun-binding inhibitory protein that can be translocated to the nucleus after interaction with presenilin-1 protein in human cerebral cortex tissue [30], and c-Jun may also contribute to the anti-proliferative effects of DMAPT in PANC-1 and MiaPaca-2 cells. However, whether and how these interactions of RPL10 and mRNAs could change the specific functions of the ribosome and the roles of c-Jun requires further investigation.

In conclusion, we have employed chemical proteomics approach to reveal the specific and direct interaction between DMAPT and RPL10 after confirming the anti-proliferative effects of DMAPT in pancreatic cancer cell lines. With the verification of the molecular interaction by MST analyses, RPL10 has been found to directly down-regulate the expression of p65 and IKKγ, which may result from the remodeling or dysfunction of ribosomal biosynthesis during the tumorigenesis.

## MATERIALS AND METHODS

### Reagents and antibodies

PTL was obtained from TAUTO biotech Co., Ltd (Shanghai, China). DMAPT was prepared from the reaction of PTL with dimethylamine, and the resulting dimethylamino analogue was converted to its water-soluble fumarate salt according to previous report [31]. The structure of DMAPT was determined by MS, 1H NMR, and >95% purity was analyzed by HPLC (Supplementary Figure 5). Antibody to RPL10 (#sc-798) was from Santa Cruz Biotechnology, Inc. (Dallas, TX, USA); IκBα (#ab32518), IKKγ (#ab125589) were from Abcam Inc. (Cambridge, MA, USA); antibody to p65 (#D14E12), STAT3 (#D3Z2G) were from Cell Signal Technology (Danvers, MA, USA). Recombinant RPL10 protein in full-length (#ab159379) was obtained from Abcam Inc. (Cambridge, MA, USA). The Fetal calf serum (FCS) and DMEM cell culture media were purchased from Life Technologies (Carlsbad, CA, USA).

### Cell cultures

PANC-1, AsPC-1, BxPC-3 and MiaPaca-2 were bought from KeyGEN Biotech (Nanjing, China) and maintained as recommended. The cells cultured in a medium containing different concentrations of DMAPT were grown for 48 h. During this period, all assays, including MTT, cell cycle, Western blotting and IP, were performed every 12 h. All cell lines used in this study were authenticated by short tandem repeat DNA finger-printing and tested for mycoplasma at GENEWIZ, Inc. (Beijing, China) before use. The reports are provided in Supplementary Figure 6.

### Cloning, expression and purification of the core-domain of RPL10

The core-domain of *RPL10* gene was PCR amplified and subcloned into pET-28a expression vector, and the vector map of RPL10 is shown in Supplementary Figure 7. The expression and purification of core domain of recombinant human RPL10 protein was carried out as previously reported [32]. Then, purified RPL10 core domain containing residues 34-182 was analyzed using 12% SDS–PAGE (Supplementary Figure 7).

### Synthesis of the affinity probe of DMAPT

DMAPT-affinity probe was prepared by coupling Michael addition to the double bond of PTL by EAH-sepharose 4B according to the synthetic route shown in Figure 1C. Briefly, 1 ml EAH-sepharose 4B (GE Healthcare Biosciences AB) was successively washed with deionized water (pH4.5), 0.5 M NaCl and PBS (pH7.4). PTL (3 mg) dissolved in 1 ml solvent (triethanolamine: alcohol 1:1) was added to the resin suspension. The mixture was stirred overnight at room temperature and dried. The resulting matrices were washed thoroughly with methanol and water, then characterized by IR spectroscopy and kept in an aqueous solution containing 20% ethanol until used in the binding experiments. The coupling rate of PTL to EAH-sepharose was 64.9% by determining the residual PTL from HPLC quantification.

### Capture and identification of specific binding proteins

According to previous method of serial affinity chromatography [33], 1 ml lysates of PANC-1 cells were gently stirred with 100 μl DMAPT-affinity probe or blank matrices (EAH-sepharose 4B) at 4 °C for approximately 40 min and then precipitated by centrifugation at 8,000 × g for 3 min. The supernatants were mixed with another 100 μl of DMAPT-affinity probe or blank matrices at 4 °C, and incubated for approximately 40 min, which was repeated for four times. The resulting resins were successively washed with lysate buffer and mixed with 40 μl of SDS loading buffer, boiled at 100 °C for 5 min, and then centrifuged for 3 min. The supernatants were subjected to SDS-PAGE. After silver staining of the gels, the protein bands were comparatively analyzed to identify the reduction in amount between each series for specific binding proteins. Individual protein band on the SDS-PAGE was excised and digested with trypsin. Tryptic peptides were analyzed on an Ultraflex II MALDI-TOF/TOF instrument (Bruker Daltonics, Bremen, Germany) in a positive-ionization mode over the *m/z* range of 700-4,000 at a resolution of 15,000 to 20,000. Protein sequences were analyzed using the peptide mass fingerprint by Mascot Search engine (version 2.4) as previously reported [34].

### Microscale thermophoresis (MST) analysis

The core-domain of RPL10 or full-length RPL10 was labeled with dye NT647 using a Monolith NT™ Protein Labeling Kit (NanoTemper Technologies, Munich, Germany) as previously described [21]. Briefly, two-fold serial dilutions of DMAPT were mixed with 15 μM of RPL10 proteins, incubated for 10 min at room temperature in 20 mM PBS (pH 8.0) and loaded in the standard Monolith NT™ capillaries (NanoTemper Technologies, Munich, Germany). Thermophoresis analysis was performed for 30 s on a NanoTemper Monolith NT™ 115 instrument (20% LED, 40% MST power) at 25 °C. Using NT Analysis software (NanoTemper Technologies, Munich, Germany), the MST curves were fitted to obtain *K*_D_ values for the direct binding affinity between RPL10 proteins and DMAPT.

### Western blotting and immunoprecipitation

Cells were incubated in the absence or presence of DMAPT for 24 h, and washed with cold PBS, then lysed for 10 min on ice with RIPA buffer. Protein samples (20 μg) were resolved on SDS-PAGE, transferred onto PVDF membranes (Millipore, USA) and incubated overnight with primary antibodies against various proteins of interest. The membranes were incubated with horseradish peroxidase (HRP)-conjugated secondary antibody for 2 h, and the bands were visualized using ELC detection kit (Millipore, USA) following the manufacturer's instructions. The immunoprecipitation was performed according the manual of Pierce Direct IP Kit (Thermo, 26148). PANC-1 lysates were pre-cleared with control beads and equal amounts of protein (1 mg) were immunoprecipitated with the antibodies of p65 or RPL10. The immunocomplexes were precipitated with AminoLink beads. Control was prepared by incubating the lysates with AminoLink agarose without coupling the antibody. Then, the samples were analyzed by Western blotting.

### Quantitative real-time PCR (qRT-PCR) analysis

Total RNA from cell lysates in all samples was extracted by TRIZOL Kit (Invitrogen, USA). Aliquots of each RNA extraction were reverse-transcribed into cDNA using the RT reagent kit (Takara, Japan). The qRT-PCR assay was performed using a LightCyler™ instrument (Roche, USA) in which the reactions were performed in a 20 μl with FastStart DNA Master SYBR Green I mix (Roche, USA). The primers used in qRT-PCR are showed in Supplementary Table 1.

### Knock-down of *RPL10* by siRNA

For transient knock-down of *RPL10*, 25 nM pools of *RPL10* targeting siRNA (Supplementary Table 2) or 25 nM pools of control (NC) constructs (Biomacs, Suzhou, China) were introduced into the cells using Lipofectamine™ 3000 (Invitrogen, USA) per the manufacturer's instructions. Cells were transfected at 50% confluency in six-well plates and seeded for MTT assay at 48 h after the transfection. Knock-downs were evaluated by western blotting of cell extracts harvested at 48 h after the transfection.

### Statistical analysis

All statistical analyses were conducted using GraphPad Prism version 6.01. Results are expressed as mean ± SD of three independent experiments. Statistical significance was determined using one-way analysis of variance (ANOVA) unless otherwise noted. *P* value <0.05 was considered statistically significant.

## SUPPLEMENTARY MATERIALS FIGURES AND TABLES


