# Supplementary material for: Cooperative down-regulation of ribosomal protein L10 and NF-κB signaling pathway is responsible for the anti-proliferative effects by DMAPT in pancreatic cancer cells
Source: Oncotarget. 2017 Mar 25;8(21):35009–18. doi: 10.18632/oncotarget.16557 (PMC5471030; doi:10.18632/oncotarget.16557)
Supplement: Supplementary file 1 [file oncotarget-08-35009-s001.pdf]

Cooperative down-regulation of ribosomal protein L10 and NF-κB signaling pathway is responsible for the anti-proliferative effects by DMAPT in pancreatic cancer cells

SUPPLEMENTARY FIGURES AND TABLES

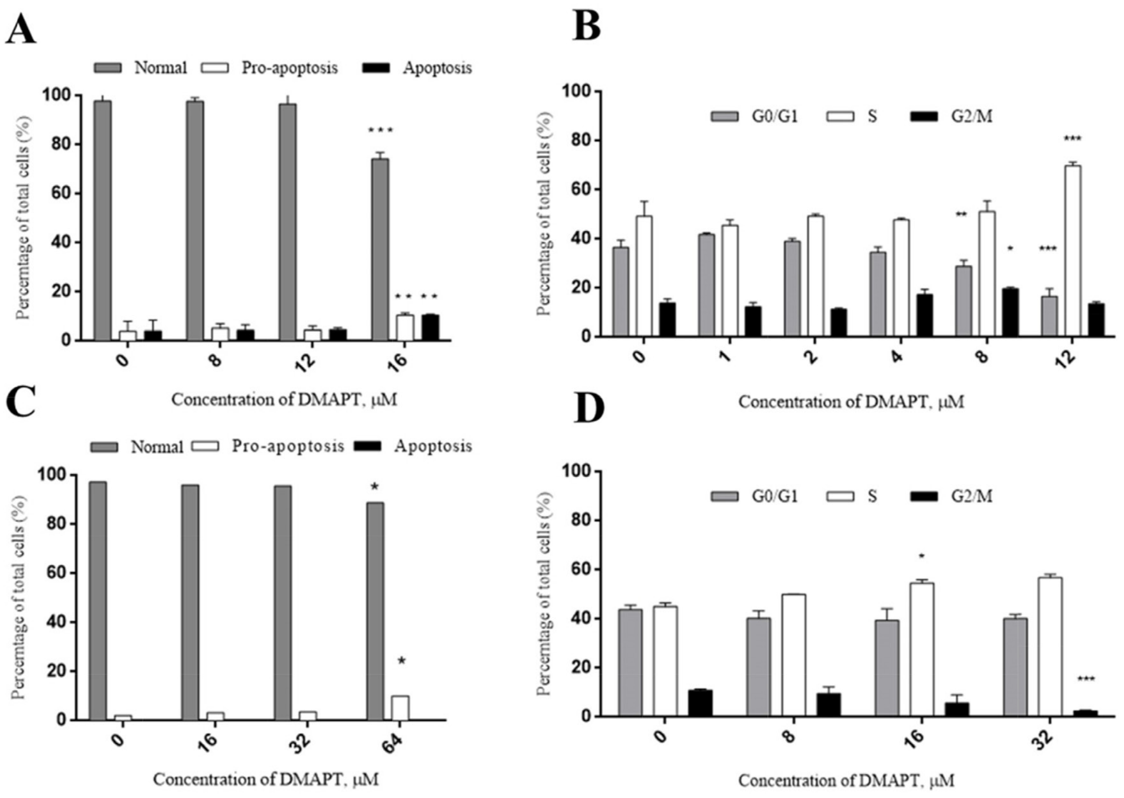

Supplementary Figure 1: Effects of DMAPT on the viability of pancreatic cancer cells. **(A)** Effects of DMAPT on apoptosis of PANC-1 cells. **(B)** Effects of DMAPT on cell cycle of PANC-1 cells. **(C)** Effects of DMAPT on apoptosis of MiaPaca-2 cells. **(D)** Effects of DMAPT on cell cycle of MiaPaca-2 cells.

A

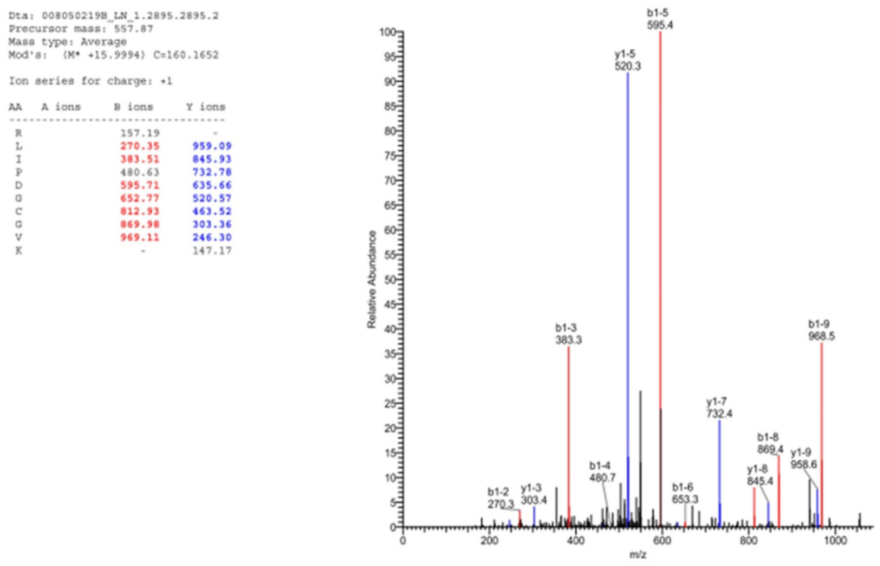

B

MGRRPARCYRYCKNKPYPKSRFCRGVPDAKIRIFDLGRKK
AKVDEFPLCGHMVSDEYEQLSSEALEAARICANKYMVKSC
GKDGFHIRVRLHPFHVIRINKMLSCAGADRLQTGMRGAFG
KPQGTVARVHIGQVIMSIRTKLQNKEHVIEALRRAKFKFPG
RQKIHISKKWGFTKFNADEFEDMVAEKRLIPDGCGVKYIPN
RGPLDKWRALHS

Sequences of Peptides	Theoretical Molecular Weight	Measured Molecular Weight
KIRIFDLGRK	990.1843	990.3790
KMLSCAGADRL	997.1025	996.8368
KMLSCAGADRL	997.1025	997.2080
KRLIPDGCGVKY	1115.3019	1114.7300
RIFDLGRK	720.8397	720.4570
RVHIGQVIMSIRT	1269.5423	1269.2610

Supplementary Figure 2: MALDI-TOF-TOF analysis of DMAPT binding protein. **(A)** Tryptic-digested peptide fragments of DMAPT binding protein; **(B)** Peptides of RPL10 identified in MS and their theoretical and detected molecular masses (Red in the amino acid sequence).

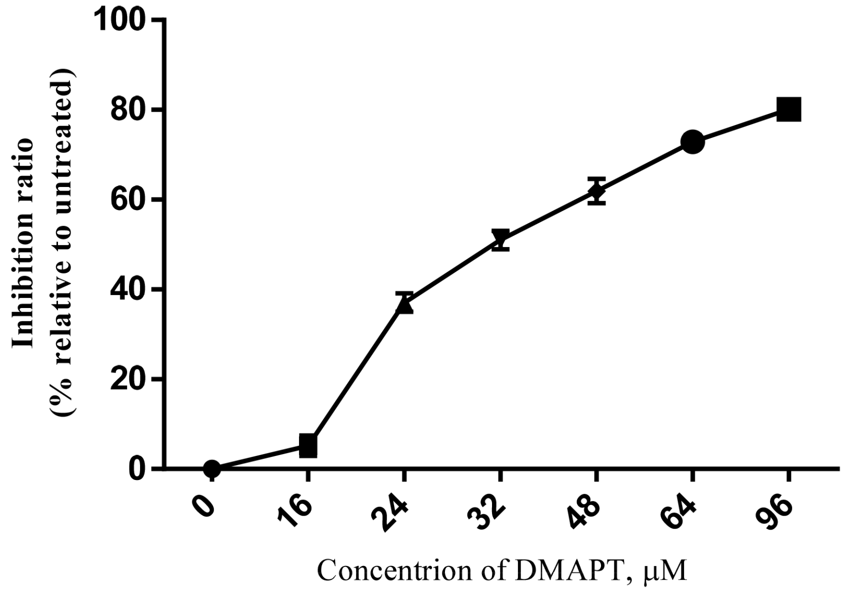

Supplementary Figure 3: The sensitivity of DMAPT against PANC-1 cells with knock-down of *RPL10*.

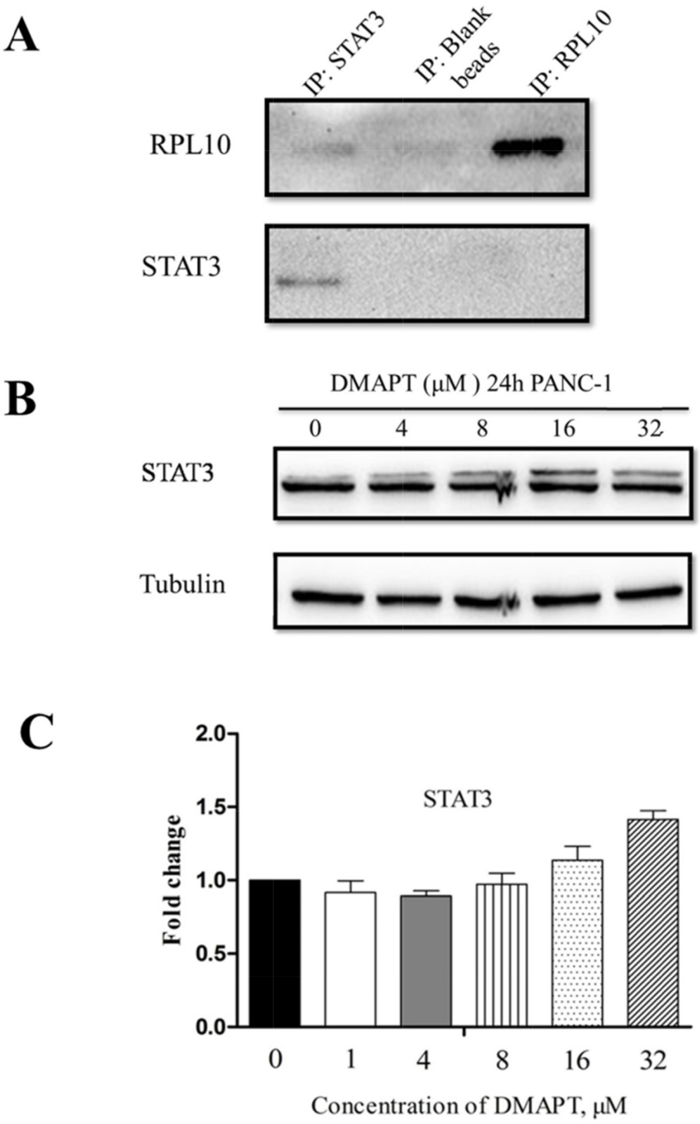

Supplementary Figure 4: The interaction of RPL10 and STAT3. **(A)** RPL10 did not interact with STAT3; **(B)** STAT3 expression treated with DMAPT in PANC-1 cells. **(C)** Changes in mRNA level of *STAT3* after DMAPT treatments of PANC-1 cells for 24 h.

A

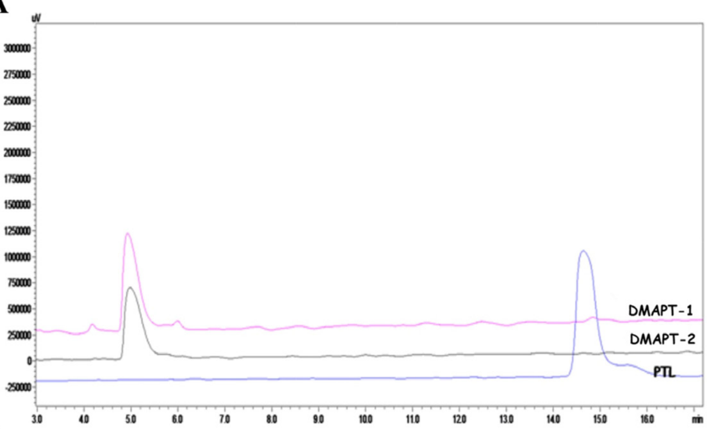

B

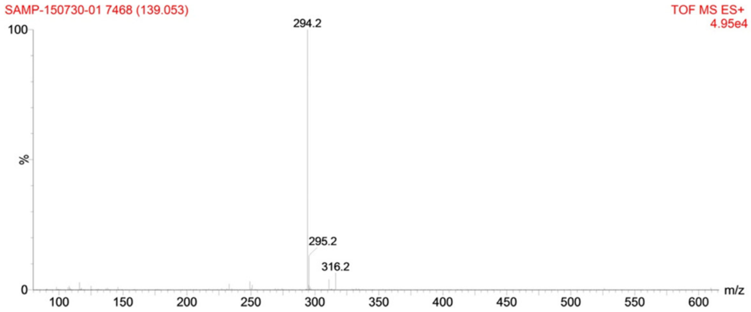

C

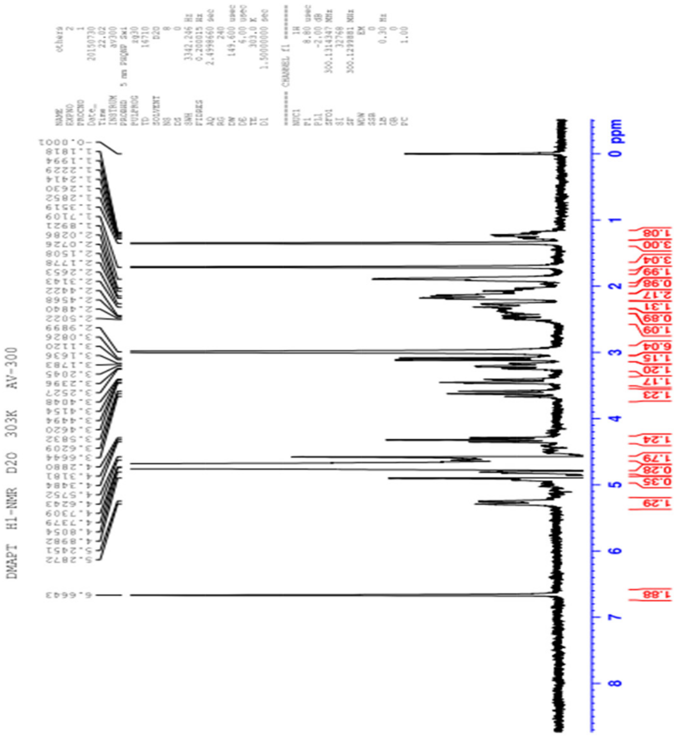

Supplementary Figure 5: MS, ^{1}H NMR and HPLC analyses of DMAPT. **(A)** The purity of DMAPT by HPLC. **(B)** Mass spectrum of DMAPT, chemical formula $C_{17}H_{27}NO_3$, calculated m/z 294.2 $[M+H]^+$, observed m/z 294.2 $[M+H]^+$. **(C)** 1H NMR (300 MHz, D_2O).

A

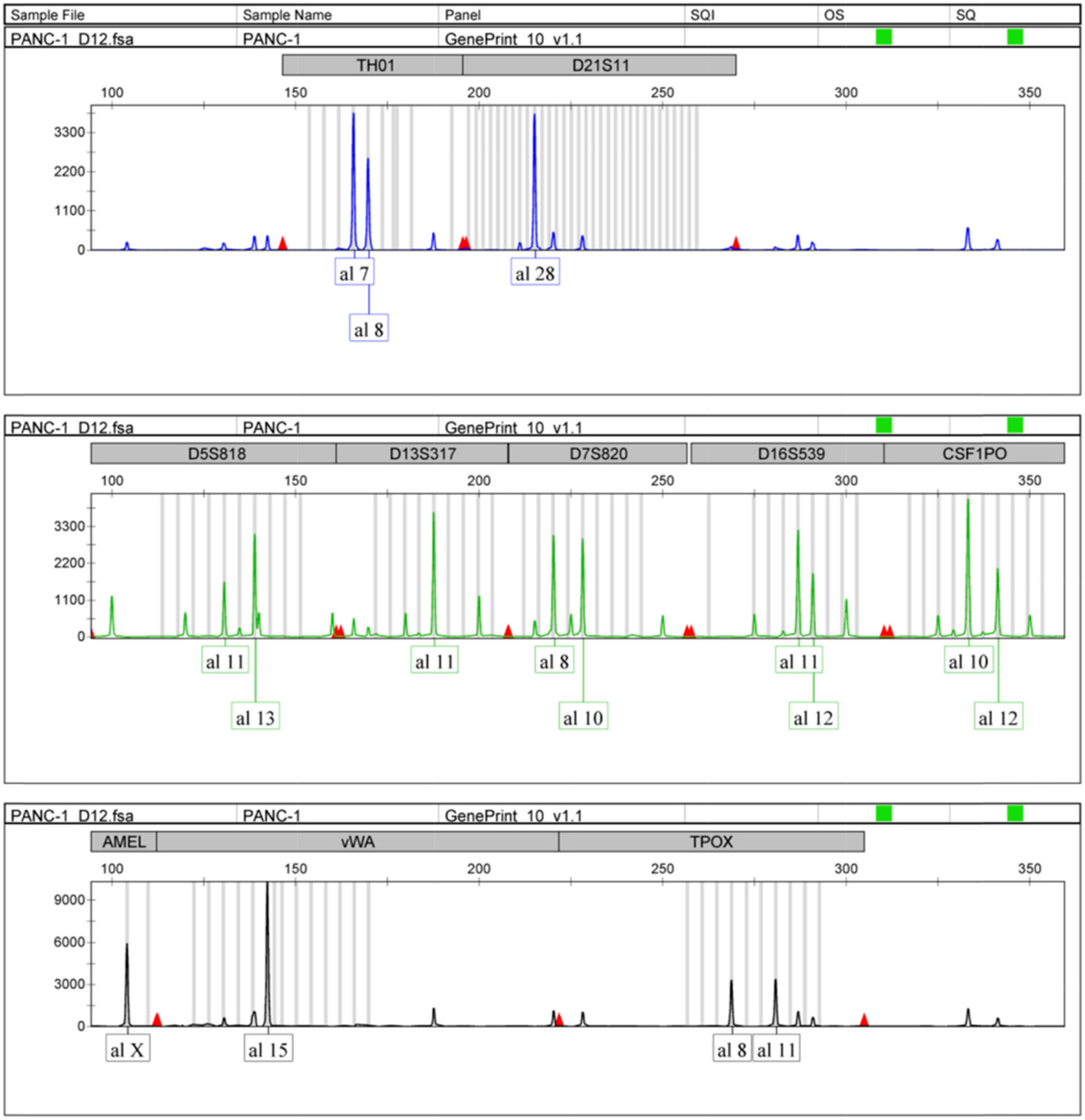
Sample File
Sample Name
Panel
SQI
OS
SQ
PANC-1_D12.fsa
PANC-1
GenePrint_10_v1.1
TH01
D21S11
100
150
200
250
300
350
3300
2200
1100
0
al 7
al 8
al 28
PANC-1_D12.fsa
PANC-1
GenePrint_10_v1.1
D5S818
D13S317
D7S820
D16S539
CSF1PO
100
150
200
250
300
350
3300
2200
1100
0
al 11
al 13
al 11
al 8
al 10
al 11
al 12
al 10
al 12
PANC-1_D12.fsa
PANC-1
GenePrint_10_v1.1
AMEL
vWA
TPOX
100
150
200
250
300
350
9000
6000
3000
0
al X
al 15
al 8
al 11

(*Continued*)

B

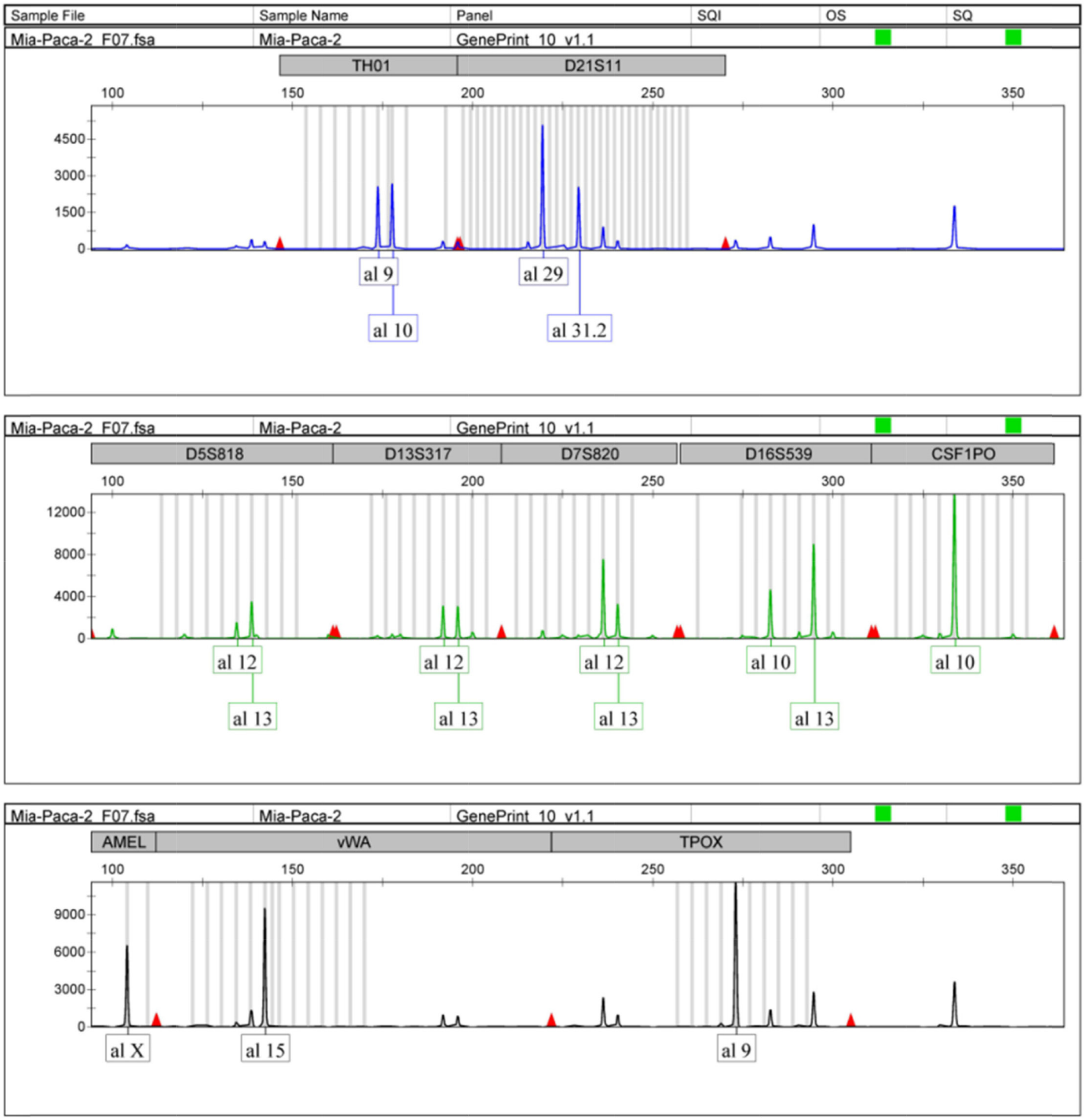
Sample File
Sample Name
Panel
SQI
OS
SQ
Mia-Paca-2_F07.fsa
Mia-Paca-2
GenePrint_10_v1.1
TH01
D21S11
100
150
200
250
300
350
4500
3000
1500
0
al 9
al 10
al 29
al 31.2
D5S818
D13S317
D7S820
D16S539
CSF1PO
12000
8000
4000
al 12
al 13
al 12
al 13
al 12
al 13
al 10
al 13
al 10
AMEL
vWA
TPOX
9000
6000
3000
al X
al 15
al 9

(Continued)

C

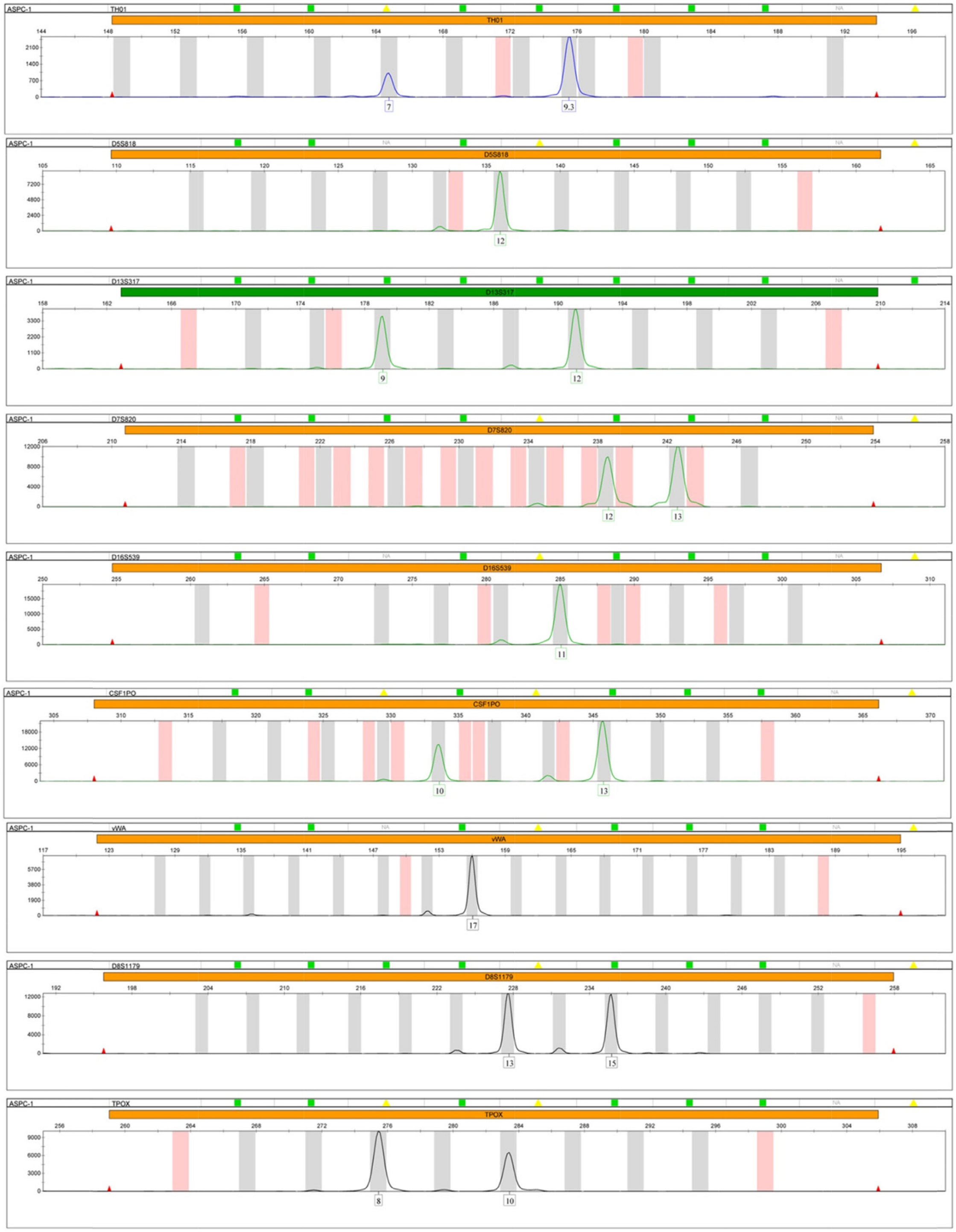
ASPC-1
TH01
7
9.3
D5S818
12
D13S317
9
12
D7S820
12
13
D16S539
11
CSF1PO
10
13
vWA
17
D8S1179
13
15
TPOX
8
10

(*Continued*)

D

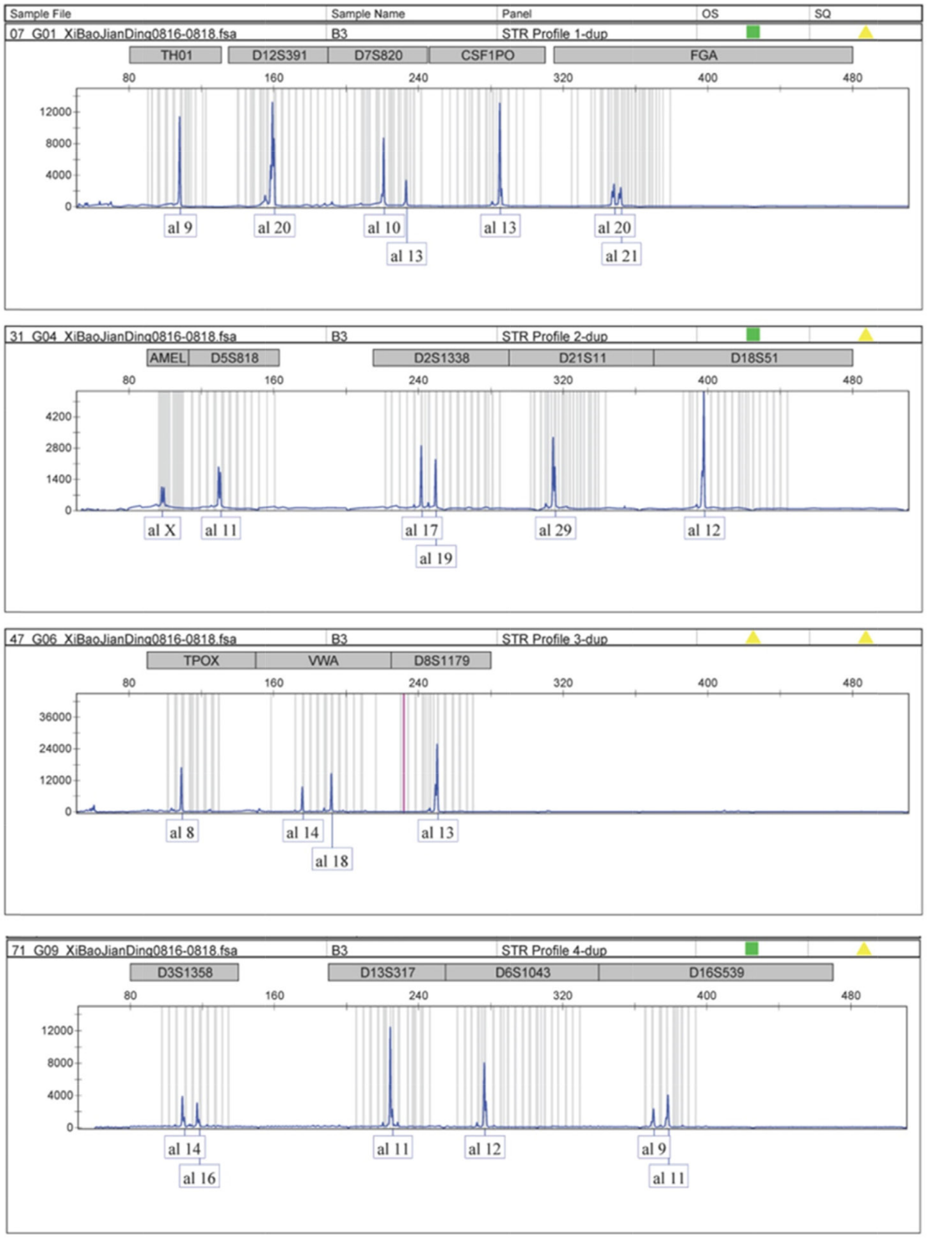

Supplementary Figure 6: STR genotyping profiles of pancreatic cancer cell lines. **(A)** PANC-1 cell line **(B)** MiaPaca-2 cell line **(C)** AsPC-1 cell line **(D)** BxPC-3 cell line.

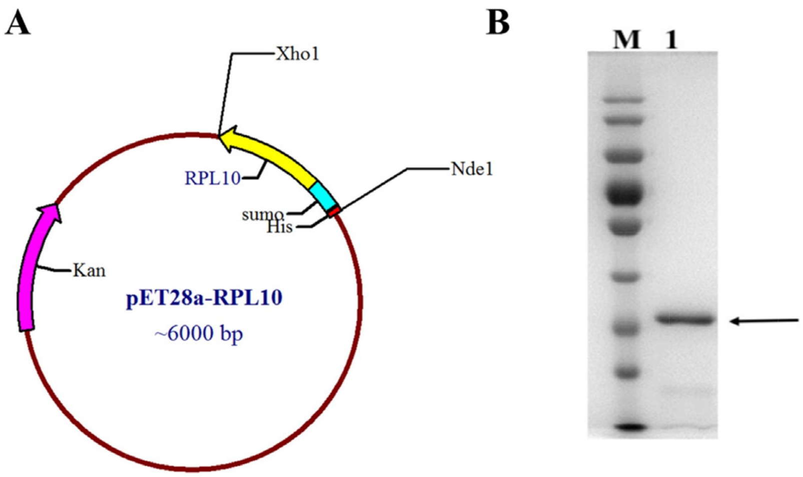

Supplementary Figure 7: Expression and purification of the core-domain of RPL10. **(A)** The expression vector of core-domain of RPL10; **(B)** SDS-PAGE of purified core-domain of RPL10. Lane M, protein molecular weight markers. Arrow shows purified core-domain of RPL10.

Supplementary Table 1: The primers used in qRT-PCR

Gene name	Forward primer	Reverse primer
β-actin	CTGGAACGGTGAAGGTGAC	AAGGGACTTCCTGTAACAATGCA
RPL10	AGCTGCAGAACAAGGAGCAT	GTGAAGCCCCACTTCTTTGA
p65	CTGCCGGGATGGCTTCTAT	CCGCTTCTTCACACACTGGAT
IKKγ	AAGAGCCAACTGTGTGAGATG	TTCGCCCAGTACGTCCTGA
STAT3	GATCCAGTCCGTGGAACCAT	ATAGCCCATGATGATTTCAGCAA

Supplementary Table 2: siRNA sequences used in the knock-down of *RPL10*

Number	positive-sense strand	antisense strand
si-a	GGCCAAGUUAUCAUGUCCAdTdT	UGGACAUGAUAACUUGGCCdTdT
si-b	GCCAAUAAGUACAUGGUAAdTdT	UUACCAUGUACUUAUUGGCdTdT
si-c	CUGAUGCCAAGAUUCGCAUdTdT	AUGCGAAUCUUGGCAUCAGdTdT
